# Beyond a dual role: how *Purpureocillium lilacinum* reprograms citrus metabolism for integrated biocontrol and growth promotion

**DOI:** 10.1002/jsfa.70954

**Published:** 2026-08-13

**Authors:** Sana Bouzembila, Kamel Charaabi, Haytham Hamden, Salma Fadhel, Nesrine Tanfouri, Meriem Msaad Guerfali

**Affiliations:** ^1^ Laboratory of Biotechnology and Nuclear Technologies, LR16CNSTN01 National Centre of Nuclear Sciences and Technologies Sidi Thabet Tunisia; ^2^ Higher Institute of Biotechnology of Sidi Thabet University of Manouba, Biotechpole Sidi Thabet Ariana Tunisia

**Keywords:** *Purpureocillium lilacinum*, *Citrus aurantium*, metabolomics, plant growth promotion, biocontrol

## Abstract

**BACKGROUND:**

Plants harbor complex microbial communities that are fundamental to their health and productivity. Among them, endophytic fungi such as *Purpureocillium lilacinum* establish mutualistic associations, offering dual benefits as plant growth promoters and biocontrol agents. However, the potential of this fungus in citrus cultivation and the metabolic dynamics of its interaction with citrus hosts remain unexplored. This study employed an integrated dual metabolomics approach to investigate the symbiosis between *P. lilacinum* and *Citrus aurantium*. It was hypothesized that the fungus would enhance plant performance through its own bioactive metabolites, and also by systemically reprogramming the host metabolism. To test this hypothesis, the *in vitro* fungal metabolome was characterized, plant growth promotion was evaluated *in vivo*, and metabolomic shifts in host tissues were analyzed.

**RESULTS:**

The results reveal a multilayered mode of action. *Purpureocillium lilacinum* stimulated plant growth directly through nutrient solubilization and phytohormone modulation. Concurrently, the fungus engaged in active metabolic cross‐talk with the host, reprogramming primary metabolism, as exemplified by the spatial redistribution of amino acids like proline and arginine, and activating secondary defense pathways. The fungal metabolome also revealed a direct biocontrol arsenal, including insect‐modulating acetophenone and the insecticidal alkaloid arecoline, underscoring its capacity for immediate antagonism.

**CONCLUSION:**

These findings provide novel insights into the biochemical mechanisms underlying *P. lilacinum*–citrus interactions. They confirm the role of the fungus in orchestrating a synergized growth‐and‐defense response in citrus, highlighting its strong potential as a sustainable, multi‐functional bioinoculant for citrus production systems. © 2026 The Author(s). *Journal of the Science of Food and Agriculture* published by John Wiley & Sons Ltd on behalf of Society of Chemical Industry.

## INTRODUCTION

Plants support complex and dynamic microbial populations in their rhizosphere, phyllosphere, and endosphere, all of which play essential roles in maintaining plant health and optimizing agricultural productivity.^1^ Among them, endophytic fungi form mutualistic associations, inhabiting plant tissues asymptomatically while conferring significant benefits.^2^ Their value derives from the prolific production of a wide spectrum of bioactive secondary metabolites, including alkaloids, carbohydrates, polyketides, terpenoids, and phenolic compounds, which exhibit antibacterial, antifungal, insecticidal, and plant growth‐promoting effects.^3^, ^4^

These fungi enhance host fitness through multiple mechanisms, solubilizing soil‐bound minerals, stimulating phytohormone biosynthesis, enhancing plant tolerance to biotic and abiotic stresses, and modulating the host's own secondary metabolism to prime defense responses.^5^, ^6^, ^7^, ^8^, ^9^ This metabolite‐mediated dialogue is fundamental to the symbiotic interaction, yet its specific pathways and outcomes remain poorly resolved for many promising endophytes.

Among diverse endophytic taxa, *Purpureocillium lilacinum* (formerly *Paecilomyces lilacinus*), a species from the Ophiocordycipitaceae family commonly found in soil and the rhizosphere, has attracted increasing attention for its biotechnological potential. Considerable interest in *P. lilacinum* stems from its dual role as a biocontrol and a plant growth‐promoting (PGP) agent.^10^, ^11^ The fungus exhibits a broad spectrum of activity, targeting root‐knot nematodes (*Meloidogyne javanica*),^12^ fungal pathogens such as *Sclerotinia sclerotiorum* and *Penicillium digitatum*,^13^, ^14^ *Verticillium dahliae*,^15^ *Phytophthora capsici*,^16^ and *Phytophthora infestans*,^17^ as well as insect pests including *Ceratitis capitata*,^18^ *Myzus persicae*,^19^ *Scirtothrips dorsalis*,^20^ and *Anastrepha ludens*.^21^ This wide‐ranging efficacy stems from a diverse arsenal of bioactive metabolites such as cell‐wall‐degrading enzymes (e.g., chitinases and proteases),^22^ antimicrobial compounds (e.g., leucinostatins and brevianamide F), and various volatile organic and phenolic compounds,^23^, ^24^ fatty acids such as lauric and myristic acid with nematicidal activity,^25^ and indole derivatives involved in activity against tobacco mosaic virus.^26^

Critically, these effects are evident *in planta*, with studies showing reduced reproduction of *Aphis gossypii* in colonized cotton plants,^27^ and increased mortality and suppressed emergence of *Bemisia tabaci* in tomato plants.^28^ Concurrently, the fungus promotes plant growth via phosphate solubilization, siderophore production, and the synthesis of phytohormones like indole‐3‐acetic acid (IAA), which collectively enhance nutrient uptake and root development.^29^, ^30^

This dual functionality positions *P. lilacinum* as a prime candidate for sustainable agriculture. However, a critical knowledge gap persists regarding a systems‐level understanding of how its metabolic arsenal directly orchestrates a reprogramming of host plant metabolism to achieve integrated growth and defense. Such metabolomic insights, although pivotal for understanding mutualisms, are notably lacking for *P. lilacinum*.

This knowledge gap is particularly relevant for perennial crops like citrus (*Citrus* spp.), a cornerstone of global horticulture with a global production exceeding 140 million t,^31^, ^32^ facing severe pressures from pathogens and abiotic stresses such as salinity.^33^, ^34^ In Tunisia, sour orange (*Citrus aurantium* L.) is a key cultivar that occupies 480 ha, with significant cultivation for its floral byproducts.^35^ The insights gained from understanding this interaction in *C. aurantium* can be extrapolated to other economically important citrus varieties, thereby addressing a critical need within a sector vital to the national economy. Sustainable strategies to enhance crop resilience are urgently needed, and bioinoculants like *P. lilacinum* offer a promising solution. Nevertheless, its potential in citrus cultivation remains unexplored, and the metabolic interplay underlying this partnership is entirely unknown.

The aim of this study was therefore to elucidate the biochemical basis of the *P. lilacinum–C. aurantium* symbiosis by applying a dual metabolomics approach. It was hypothesized that *P. lilacinum* promotes growth and exerts biocontrol not only through its own bioactive metabolites but also by systemically reprogramming the host plant's primary and secondary metabolism. To test this hypothesis, the study (1) characterized the *in vitro* fungal metabolome and growth‐promoting traits, (2) evaluated growth enhancement in inoculated plants under greenhouse conditions, and (3) analyzed the consequent metabolomic shifts in host tissues to establish direct links between fungal activity, host metabolic reprogramming, and phenotypic improvements.

## MATERIALS AND METHODS

Figure 1 summarizes the overall experimental design and workflow adopted in this study.

**Figure 1:**
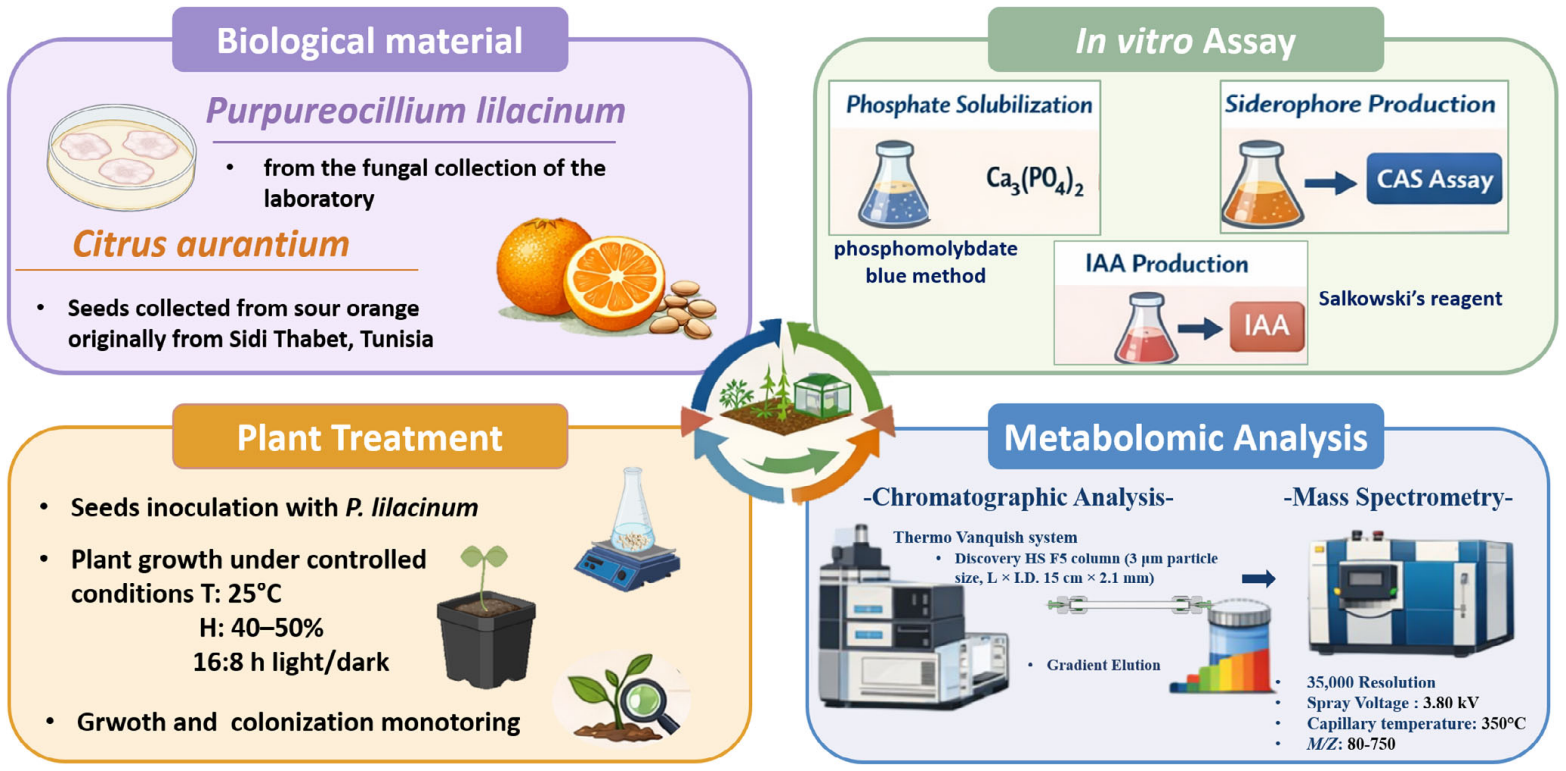
Schematic overview of the experimental design and analytical workflow, including biological material sources, *in vitro* plant growth‐promoting (PGP) assays, plant inoculation and growth conditions, and untargeted liquid chromatography–tandem mass spectrometry (LC–MS/MS)‐based metabolomic analysis.

### Biological material sources

The *P. lilacinum* strain used in this study was obtained from the fungal collection of the Laboratory of Biotechnology and Nuclear Technology at the National Centre of Nuclear Sciences and Technology (CNSTN). The strain had been isolated from soil samples in a citrus orchard in Nabeul governorate, Tunisia (36° 27′ 21″ N, 10° 44′ 15″ E, altitude 14 m).^18^

Seeds of sour orange (*C. aurantium* L.) were extracted from mature fruits collected in the Sidi Thabet region of Ariana governorate, Tunisia (Plus Code: W26R + 39M, Sidi Thabet).

### Plant growth traits

#### Quantitative estimation of phosphate solubilization

The ability of *P. lilacinum* to solubilize inorganic phosphate was assessed using tricalcium phosphate Ca₃(PO₄)_2_ (CAS 7758‐87‐4, Fisher chemical, Waltham, MA, USA) as an insoluble phosphorus source in Pikovskaya medium broth.^29^ A 50 μL aliquot of a conidial suspension (1 × 10^5^ conidia mL^−1^) was inoculated into 120 mL of broth. Control flasks received 50 μL of sterile distilled water instead of the fungal suspension. All experiments were conducted with three independent biological replicates. All flasks were incubated for 10 days at 28 ± 1 °C under a 12 h light/dark cycle with agitation at 150 rpm. At 48 h intervals, 10 mL aliquots were aseptically collected from each flask. The samples were centrifuged at 6000 × *g* for 10 min and the supernatant was filtered through a 0.45 μm nylon membrane filter to remove mycelial fragments and residual solids. The concentration of solubilized phosphate in the culture filtrate was quantified using the phosphomolybdate blue method,^36^ measuring absorbance at 820 nm. A standard curve was generated using known concentrations of KH_2_PO_4_ (CAS: 7778‐77‐0, Sigma Aldrich, St. Louis, MO, USA) (concentration = [absorbance + 0.5042] / 0.0683, *R* ^2^ = 0.9233).

#### Quantitative estimation of siderophore production

Siderophore production by *P. lilacinum* was quantified in an iron‐deficient medium (SIM), prepared as described by Alexander and Zuberer,^37^ with minor modifications. Briefly, 30.2 g of piperazine‐*N*,*N*′‐bis(2‐ethanesulfonic acid) (PIPES) buffer (CAS: 5625‐37‐6, Thermo Scientific) was dissolved in 750 mL of a salt solution containing KH_2_PO₄ (0.3 g) (CAS: 7778‐77‐0, Sigma Aldrich), NaCl (0.5 g) (CAS: 7647‐14‐5, Sangon Biotech, Shanghai, China), and NH₄Cl (1.0 g) (CAS: 12125‐02‐9, Loba Chemie, Mumbai, India). The pH was adjusted to 6.8, and the final volume was brought to 800 mL with distilled water (solution 1). A Fe‐free trace elements solution (solution 2) was prepared with glucose (2.0 g) (CAS: 50‐99‐7, Bio Basic Inc, Markham, Canada), mannitol (2.0 g) (CAS: 69‐65‐8, Loba Chemie), MgSO₄·7H_2_O (CAS: 10034‐99‐8, Sigma Aldrich) (493 mg), CaCl_2_ (11 mg) (CAS: 10043‐52‐4, Bio Basic Inc), MnSO₄·H_2_O (1.17 mg) (CAS: 10034‐96‐5, Loba Chemie), H₃BO₃ (1.4 mg) (CAS: 10043‐35‐3, Scharlau, Barcelona, Spain), CuSO₄·5H_2_O (0.04 mg) (CAS: 7758‐99‐8, Bio Basic Inc), ZnSO₄·7H_2_O (1.2 mg) (CAS: 7446‐20‐0, Bio Basic Inc), and Na_2_MoO₄·2H_2_O (1.0 mg) (CAS: 10102‐40‐6, Loba Chemie) in 70 mL distilled water. Casamino acids (Catalog number: CB3060 Bio Basic) (10%, w/v) were prepared separately (solution 3) and filter sterilized (0.2 μm). Solutions 1 and 2 were autoclaved, then mixed with solution 3, and the volume was adjusted to 1 L with sterile distilled water.

For fungal cultivation, 100 mL of SIM was dispensed into sterile Erlenmeyer flasks and inoculated with four 6 mm diameter agar plugs of 7‐day‐old *P. lilacinum* cultures, with uninoculated flasks serving as controls. All experiments were performed in triplicate (*n* = 3 independent biological replicates). Samples were collected at 48, 96, and 144 h post‐inoculation, centrifuged, and filtered (0.2 μm). Siderophore detection was performed using the chrome azurol S (CAS) assay, in which culture supernatants were mixed with an equal volume of CAS reagent (CAS: 1667‐99‐8, Sigma Aldrich). The absorbance was measured at 630 nm after 48, 96 and 144 h and the percentage of siderophore units was calculated as (*A*_r_ − *A*_s_) / *A*_r_ × 100, where *A*_r_ is the reference absorbance (CAS reagent with sterile medium) and *A*_s_ is the sample absorbance.^38^

#### Quantitative estimation of IAA production

The tryptophan‐dependent production of IAA by *P. lilacinum* was assessed in Czapek Dox broth supplemented with 0.1% (w/v) l‐tryptophan (CAS: 73‐22‐3, Bio Basic Inc). This supplementation was used to evaluate the strain's biosynthetic potential through the tryptophan‐dependent pathway. Flasks containing 120 mL of medium were inoculated with 50 μL of conidial suspension (1 × 10^5^ conidia mL^−1^), while control flasks received an equivalent volume of sterile distilled water. All experiments were conducted in triplicate (*n* = 3 independent biological replicates). Flasks were incubated for 10 days at 28 ± 1 °C under a 12 h light/dark cycle with agitation at 180 rpm. At 48, 120, and 168 h post‐inoculation, 2 mL aliquots were aseptically collected, centrifuged at 6000 × *g* for 10 min and the supernatant was used for analysis. Indole‐3‐acetic acid in the cell‐free supernatant was quantified colorimetrically by mixing it with Salkowski's reagent (1 mL of 0.5 mol. L^−1^ FeCl_3_ (CAS: 7705‐08‐0, SRLchem, Maharashtra, India) in 50 mL of 35% HClO_4_ (CAS: 7601‐9‐3, VWR International, Fontenay‐sous‐bois, France))  in a 1:2 (v/v) ratio. After a 30 min incubation in the dark, the absorbance of the developed pink‐to‐red color was measured at 530 nm. The IAA concentration was determined using a standard curve generated with pure IAA (CAS: 87‐51‐4, Bio Basic Inc) (absorbance = 0.064 × IAA concentration + 0.1818; *R* ^2^ = 0.9899).

### Plant treatment

A conidial suspension of *P. lilacinum* was prepared from 7‐day‐old sporulating cultures. The culture surface was flooded with sterile distilled water containing Tween 80 (0.1% v/v, Difco™) and the conidia were harvested by gentle scraping. The resultant suspension was filtered through several layers of sterile gauze to remove mycelial debris. The conidial concentration was determined using a hemocytometer and adjusted to 1 × 10^7^ conidia mL^−1^ with sterile water plus 0.1% Tween 80. Conidial viability was assessed prior to the bioassays using a germination test. Briefly, a low concentration of conidia was suspended in 0.1% Tween 80, and a 100 μL aliquot of the suspension was spread onto PDA plates and incubated at 25 °C for 24 h.

Germination was evaluated by examining three randomly selected groups of 100 conidia. A conidium was considered germinated when the germ tube length was at least twice the diameter of the conidium. Germination rates were calculated as the mean of three replicate counts and exceeded 90% on average. Seeds of *C*. *aurantium* were surface sterilized by immersion in 2.5% sodium hypochlorite for 3 min and 70% ethanol for 2 min, followed by three rinses in sterile distilled water. After air‐drying on sterile filter paper for 30 min, seeds were divided into two treatment groups. Seeds in the inoculation group were soaked in the prepared conidial suspension (1 × 10^7^ conidia mL^−1^) for 24 h at 25 °C under agitation at 100 rpm, while control seeds were soaked in sterile water under identical conditions. Subsequently, each seed was sown in a plastic pot containing sterile potting soil. Immediately after sowing, each pot received a soil drench with 10 mL of the corresponding conidial suspension or sterile distilled water plus 0.1% Tween 80. Two additional soil inoculations were performed at 7 and 14 days post‐sowing.

Pots were maintained under controlled greenhouse conditions (22 ± 3 °C, 40% to 50% relative humidity, and a photoperiod of 16:8 h light/dark). Plants were irrigated with sterile distilled water as needed, without the addition of fertilizer during the course of the experiment.

#### Confirmation of plant colonization

Colonization of *C. aurantium* by *P. lilacinum* was confirmed by polymerase chain reaction (PCR) analysis targeting fungal‐specific genomic DNA.^27^ Leaf samples were collected from both treated and control plants, flash‐frozen in liquid nitrogen and ground to a fine powder. Total genomic DNA was extracted using a cetyltrimethylammonium bromide (CTAB) method.^39^ The PCR analysis was performed using primers specific to *P. lilacinum* (forward primer: 5′CTCAGTTGCCTCGGCGGGAA3′, and reverse primer 3′GTGCAACTCAGAGAAGAAATTCCG 5′).^40^ The reaction conditions consisted of an initial denaturation at 95 °C for 2 min, followed by 35 cycles of 94 °C for 1 min, 61 °C for 1 min, and 72 °C for 1 min, with a final extension at 72 °C for 5 min. The resulting amplicons were visualized by electrophoresis on a 2% (w/v) agarose gel.

#### Measurement of plant growth parameters

Plant growth was assessed using four biological replicates (*n* = 4) by measuring several biometric parameters at harvest. Shoot and root fresh weights were recorded immediately. For dry weight determination, tissues were oven‐dried at 70 °C for 48 h until a constant mass was achieved. Plant height was measured from the root collar to the apical meristem. The number of fully expanded leaves present on the main stem was counted.

#### Quantification of photosynthetic pigments

The chlorophyll *a* (Chl a), chlorophyll *b* (Chl b), total chlorophyll (Chl t), and carotenoid content in leaf tissue was quantified as described by Lichtenthaler.^41^ Briefly, 100 mg of fresh leaf tissue was homogenized in 10 mL of 80% (v/v) acetone (CAS: 67‐64‐1, Loba Chemie). The homogenate was centrifuged at 5000 × *g* for 5 min, and the absorbance of the supernatant was measured at 663, 646, and 470 nm. All measurements were performed using four biological replicates (*n* = 4). Pigment concentrations were calculated using the following equations:

$$\mathrm{Chl}\;a\;\left(\mathrm{\mu g}/g\;\mathrm{FW}\right)=\frac{\left(12.25\left(A663\right)-2.79\;\left(A646\right)\right)\times V}{M}$$

$$\mathrm{Chl}\;\;b\;\left(\mathrm{\mu g}/g\;\mathrm{FW}\right)=\frac{\left(21.50\;\left(A646\right)-5.1\;\left(A663\right)\right)\times V}{M}$$

$$\mathrm{Chl}\;t\;\left(\mathrm{\mu g}/g\;\mathrm{FW}\right)=\frac{\left(7.15\;\left(A663\right)+18.71\;\left(A646\right)\right)\times V}{M}$$

$$\text{carotenoid}\left(\mathrm{\mu g}/g\;\mathrm{FW}\right)=\frac{\left(\left(1000\times A470-1.8\times \mathrm{chl}\;a-85.02\times \mathrm{chl}\;b\right)/198\right)\times V}{M}$$

where A663, A646, and A470 represent the absorbance values measured at 663, 646, and 470 nm, respectively; V represents the volume of acetone extract (mL); M represents the fresh weight of leaf tissue (g); and FW represents fresh weight.

#### Statistical analyses

All experiments were performed using independent biological replicates: *n* = 4 for growth and pigment assays, and *n* = 3 for all other biochemical assays. Data normality was checked using a Shapiro–Wilk test, at an alpha significance level of 0.05, prior to any data analysis. For *in vitro* assays (solubilization of inorganic phosphorus sources, production of siderophores, and the production of IAA), differences among treatments over time were analyzed using one‐way analysis of variance (ANOVA), followed by an appropriate *post hoc* test. Differences in plant growth parameters (dry weight, plant height, chlorophyll, and carotenoids) between inoculated and control groups were assessed using a Student's *t*‐test. All analyses were performed using GraphPad Prism (version 8.0 software for Windows).

### Metabolomic profiling

#### Metabolite extraction from *P. lilacinum*

Intracellular metabolites were extracted from six independent biological replicates (*n* = 6) of *P. lilacinum* using the cold acetone method. A 1 mL aliquot of a conidial suspension (10^8^ conidia mL^−1^) was inoculated into liquid malt extract medium and incubated for 14 days at 25 °C. The resulting mycelial mat was removed by filtration through sterile gauze. The conidia were then recovered by centrifugation at 5000 × *g* for 15 min. The pellet was resuspended in 1 mL of cold acetone (−20 °C) and incubated overnight at −20 °C. Finally, the acetone was removed at room temperature under a fume hood.

#### Metabolite extraction from *C. aurantium*

Leaves, stems, and roots were lyophilized and ground into a fine powder using liquid nitrogen. For each plant organ, six independent biological replicates (*n* = 6) were analyzed. Metabolites were extracted from 50 mg of powdered tissue using 1 mL of 50:50 methanol/water (MeOH/H_2_O) solution, sonicated in an ultrasonic bath at 4 °C for 20 min. Samples were centrifuged at 10 000 × *g* for 10 min, and the supernatant was collected. The remaining pellet was re‐extracted with 1 mL of pure methanol, sonicated, and centrifuged under the same conditions. The supernatants from both extractions were pooled and dried using a SpeedVac centrifugal vacuum concentrator (Thermo Fisher Scientific, Waltham, MA, USA).

#### Sample preparation for liquid chromatography‐mass spectrometry analysis

Dried metabolite extracts from plant and fungal materials were prepared for liquid chromatography‐mass spectrometry (LC–MS) analysis following distinct protocols. For plant metabolite extraction, dried extracts were reconstituted in 200 μL of LC‐grade water by pipetting to ensure complete dissolution. The mixture was centrifuged at 20 000 × *g* for 10 min at 4 °C. A 10 μL aliquot was then resuspended in 90 μL of LC‐grade water. The final 100 μL solution was transferred into LC vials for LC–MS analysis.

For fungal metabolite extraction, samples were prepared following the STAR protocol. Dried fungal extracts were homogenized in 750 μL of a pre‐cooled solvent mixture (methanol/water/formic acid, 66:33:1, v/v/v) in a screw‐cap tube containing 1.0 mm glass beads, using a vortex mixer for 2 min. After vortexing, the mixture was centrifuged at 20 000 × *g* for 10 min at 4 °C, and 600 μL of the resulting supernatant was collected and dried overnight in a SpeedVac. The dried extracts were reconstituted in 100 μL of LC‐grade water, mixed thoroughly, and centrifuged again at 20 000 × *g* for 10 min at 4 °C. The final supernatant was transferred into LC vials for analysis. Solvent blanks were included in the analytical sequence to detect potential background contaminants and ensure data reliability.

#### Untargeted metabolomic profiling by liquid chromatography–tandem mass spectrometry instrumentation

Chromatographic separation was performed with a Thermo Vanquish system (Thermo Fisher Scientific) equipped with a high‐performance liquid chromatography (HPLC) Discovery HS F5 column (3 μm particle size, length × internal diameter 15 cm × 2.1 mm, catalog number 567503‐U; Sigma‐Aldrich) maintained at 40 °C. Mobile phase A consisted of 0.1% formic acid (catalogue number 33015; Sigma‐Aldrich, St. Louis, Missouri, USA.) in ultra‐performance liquid chromatography (UPLC)‐grade water (catalog number 0023214102BS; Biosolve BV, Valkenswaard, The Netherlands), and mobile phase B consisted of 0.1% formic acid in UPLC‐grade acetonitrile (catalog number 0001207802BS; Biosolve BV). The elution gradient was applied as follows: 0% B at 0.00 min, 0% B at 2.00 min, 25% B at 5.00 min, 35% B at 11.00 min, 95% B at 15.00 min, 95% B at 20.00 min, 0% B at 20.10 min, and 0% B at 25.00 min. The column temperature was set at 40 °C, the flow rate was 0.250 mL min⁻¹, and the injection volume was 0.5 μL for each run. General metabolite profiling was performed on a Q Exactive Plus mass spectrometer (Thermo Fisher Scientific, Bremen, Germany) in positive ionization mode using a full scan (80 to 750 *m/z*) at 35 000 resolution, followed by data‐dependent acquisition (DDA) MS/MS analysis. Source‐parameter settings were as follows: spray voltage, 3.80 kV; gas temperature, 200 °C; auxiliary gas flow, 10 arbitrary units; capillary temperature, 350 °C; and sheath gas flow, 40 arbitrary units.

The raw data were processed using Compound Discoverer 3.3 software (Thermo Fisher Scientific) for baseline filtering, peak alignment, peak picking, and normalization to generate peak intensities for retention time (RT) and *m/z* data pairs. Metabolites were identified using the mzCloud, mzVault, and MassList databases. The processed data, including RT–m/z variables and their peak areas, were exported from the software for subsequent statistical analysis.

Metabolomics profiling was performed using the Montpellier Alliance for Metabolomics and Metabolism Analysis (MAMMA) platform (https://www.biocampus.cnrs.fr/index.php/fr/plateforme‐biocampus/plateformes‐technologiques/428‐mamma‐2).

#### Statistical analysis of metabolomics data

Metabolomic data were expressed as relative abundances based on normalized peak areas, because this study relied on untargeted LC–MS/MS profiling rather than absolute quantification. Statistical analysis of processed LC–MS/MS data was performed using MetaboAnalyst (v6.0). Data were normalized by sum, log₁₀‐transformed, and scaled using Pareto scaling prior to multivariate and univariate analyses.

Orthogonal partial least squares discriminant analysis (OPLS‐DA) was performed to visualize metabolic differences between *P. lilacinum*‐treated and control plant groups. Model validity was evaluated using leave‐one‐out cross‐validation (LOOCV) and permutation tests, with performance assessed based on accuracy, the multiple correlation coefficient (*R* ^2^ *Y*), and the cross‐validated predictive ability (*Q* ^2^ *Y*). Variable importance in projection (VIP) scores were calculated from the OPLS‐DA model to identify the metabolites contributing most to group discrimination, considering metabolites with VIP higher than 1 as the most influential.

Univariate statistical analysis was performed using one‐way analysis of variance (ANOVA), with significance set at *P* < 0.05. Fold change (FC) and log_2_(FC) values were computed to assess the direction and magnitude of metabolite variation between groups. Volcano plots and hierarchical clustered heatmaps were generated to visualize significantly altered metabolites and their abundance patterns across different plant tissues and treatments.

## RESULTS

Successful colonization of citrus plants by *P. lilacinum* was confirmed using PCR amplification. A specific amplicon corresponding to the expected fungal DNA fragment was detected in inoculated plants, whereas no amplification was observed in non‐inoculated controls. These results confirm the successful establishment of *P. lilacinum* within plant tissues. The corresponding gel electrophoresis is provided in Supporting Information, Fig. S1.

### Plant growth promoting traits of *Purpureocillium lilacinum*

The ability of *P. lilacinum* to solubilize inorganic phosphate in liquid culture was evaluated over an 8 day incubation period and compared with an untreated control (Fig. 2). As Fig. 2(A) shows, phosphate solubilization in the control remained minimal and relatively constant, with no significant variation over time. In contrast, *P. lilacinum* exhibited a time‐dependent increase in phosphate solubilization capacity, which increased gradually from day 2 and peaked at approximately 213–250 mg L^−1^ on day 8 (*P* < 0.001), indicating a strong and progressive enhancement of solubilization activity.

**Figure 2:**
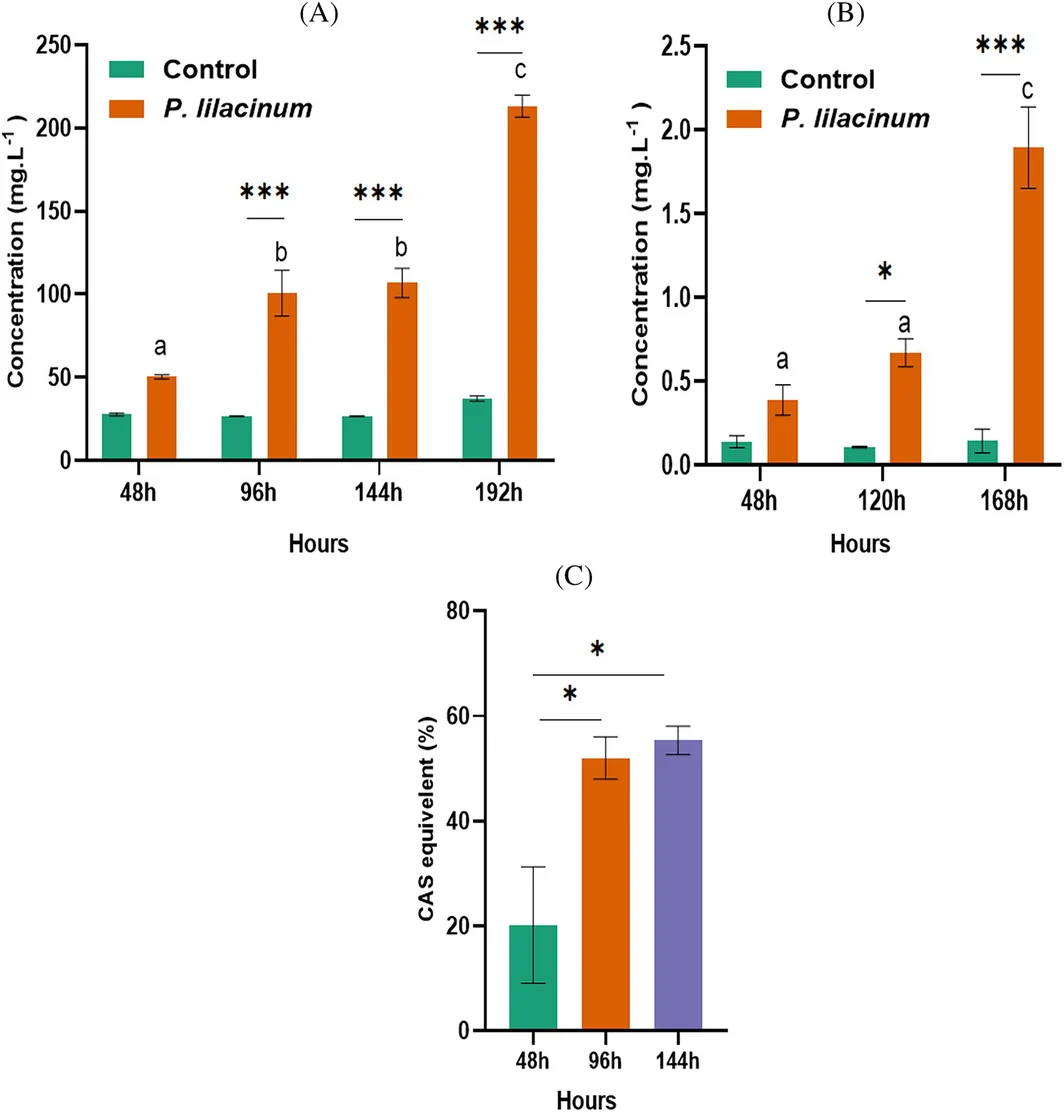
(A) Solubilization of Ca₃(PO₄)_2_, (B) siderophore production, and (C) indole‐3‐acetic acid (IAA) production by *Purpureocillium lilacinum*. Data are presented as means ± SE (*n* = 3). Statistical analysis was performed using one‐way analysis of variance (ANOVA) followed by Tukey's multiple comparison test (*P* < 0.05). Different letters indicate significant differences among time points within the same treatment, and asterisks indicate significant differences between treatments and control at the same time point (*P < 0.05; ***P < 0.001).

Siderophore production, expressed as CAS equivalents, was also time dependent. Levels were low at 24 h (20%), but increased significantly by 96 h (51.9%, *P* = 0.0415). Production appeared to stabilize thereafter, with no significant difference between levels at 96 h and 144 h (55.3%, *P* = 0.9392) (Fig. 2(B)).

The capability of *P. lilacinum* to synthesize the phytohormone IAA was also investigated using 0.1% l‐tryptophan. Indole levels did not differ significantly between the control and *P. lilacinum* at 48 h (*P* = 0.199) (Fig. 2(C)). A significant induction was observed at 120 h (*P* = 0.046), culminating in a highly significant increase at 168 h (*P* < 0.001), indicating a progressive induction of indole production by *P. lilacinum* over time.

### Plant growth parameters

Inoculation with *P. lilacinum* significantly enhanced vegetative growth of *C. aurantium* under greenhouse conditions (Fig. 3). The fresh weight of treated plants was 78% higher than that of controls (7838 ± 339.8 mg vs 4416 ± 159.9 mg; *t* = 9.116, *P* < 0.0001). Shoot fresh weight was also doubled (7120 ± 369.7 mg vs 3784 ± 130.5 mg; *t* = 8.508, *P* = 0.0001), and shoot dry weight showed a substantial increase (1955 ± 137.6 mg vs 1119 ± 36.81 mg; *t* = 5.875, *P* = 0.0011). Although root fresh and dry weights increased modestly by 2% to 15%, these changes were not statistically significant (root fresh: *t* = 1.695, *P* = 0.189; root dry: *t* = 1.974, *P* = 0.096).

**Figure 3:**
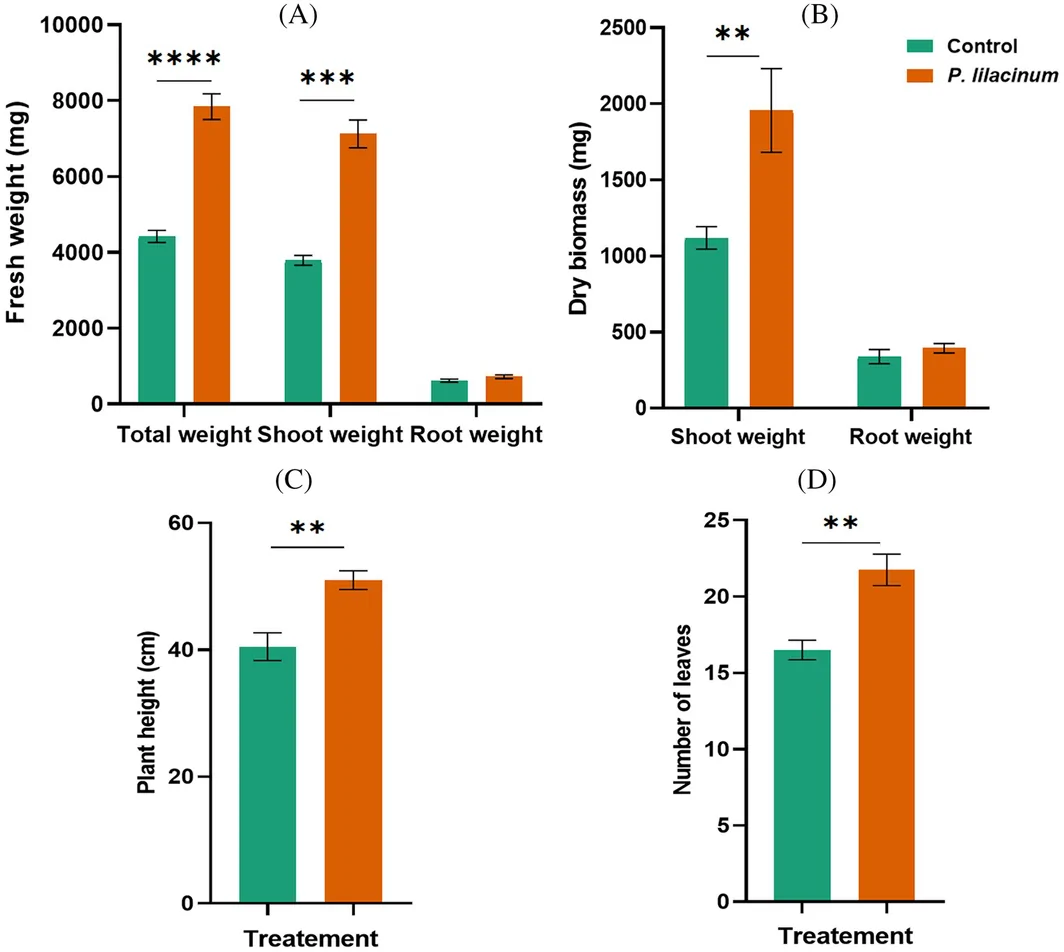
Growth promotion of *Citrus aurantium* by *Purpureocillium lilacinum*: (A) shoot fresh weight; (B) shoot dry weight; (C) plant height, and (D) leaf number measured 1 year after transplantation. Data are presented as means ± SE (*n* = 4). Statistical analysis was performed using Student's *t‐test* one‐way analysis of variance (ANOVA) followed by Tukey's multiple comparison test (*P* < 0.05). Significant differences are indicated as **P < 0.01; ***P < 0.001; ****P < 0.0001.

Beyond biomass, treated plants exhibited significant improvements in other growth parameters: plant height increased by 20% (*P* < 0.001), and the number of leaves increased by 30% (*P* < 0.01) compared with the control plants.

### Enhancement of photosynthetic pigments in *C. aurantium* by *P. lilacinum*

To investigate the impact of *P. lilacinum* inoculation on photosynthetic performance and chloroplast function in *C. aurantium* seedlings, Chl a, Chl b, Chl t, and carotenoid concentrations were measured (Fig. 4). Inoculated plants exhibited a pronounced enhancement of all pigments compared with the uninoculated control. The Chl a content increased by 214%, from 177.2 μg g^−1^ FW in controls to 557.5 μg g^−1^ FW in treated plants (*P* < 0.001), and Chl b levels nearly tripled in the inoculated group (150.0 versus 445.9 μg g^−1^ FW, *P* < 0.001). This led to a substantial rise in total chlorophyll, exceeding 1000 μg g^−1^ FW in treated plants versus 327.1 μg g^−1^ FW in controls (*P* < 0.001). Carotenoid concentrations also doubled, from 350.3 μg g^−1^ FW in controls to 733.0 μg g^−1^ FW following inoculation (*P* < 0.001).

**Figure 4:**
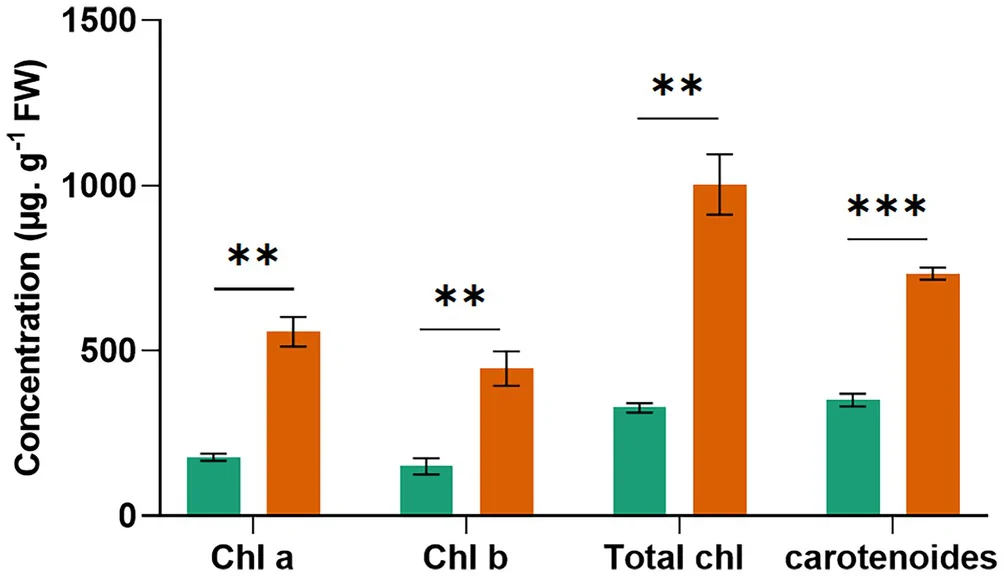
Effect of *Purpureocillium lilacinum* inoculation on photosynthetic pigments (chlorophyll a, chlorophyll b, total chlorophyll, and carotenoids) in *Citrus aurantium* 1 year after transplantation. Data are presented as means ± SE (*n* = 4). Statistical differences were assessed using Student's *t*‐test (*P < 0.05). Significant differences are indicated as **P < 0.01; ***P < 0.001.

### *Purpureocillium lilacinum* metabolite profiling

Metabolomic profiling was carried out using LC–MS/MS in positive ion mode, enabling the identification of metabolites based on retention time, accurate mass‐to‐charge ratio (*m/z*), and MS/MS fragmentation patterns (Fig. 5). In total, 70 metabolites were detected (Supporting Information, Table S1), and classified into nine distinct chemical groups (Fig. 5(A)). Applying the Metabolomics Standards Initiative (MSI) guidelines, all reported metabolites achieved MSI Level 2 confidence (putative annotation based on spectral library MS/MS matches), as detailed in Supporting Information, Table S1.

**Figure 5:**
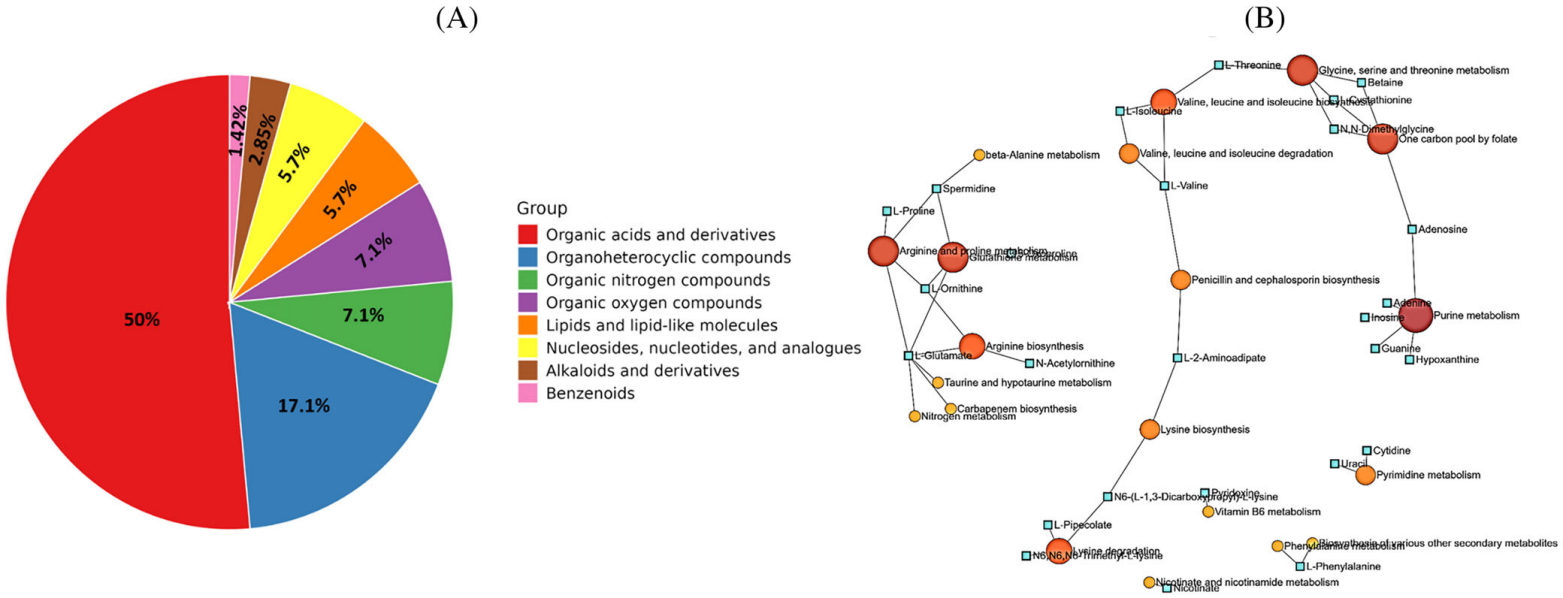
Metabolomic profile of *Purpureocillium lilacinum*. (A) Classification of metabolites identified by untargeted liquid chromatography–tandem mass spectrometry (LC–MS/MS) into major chemical classes. (B) Pathway enrichment analysis of significantly overrepresented metabolites. Data are presented from six biological replicates (*n* = 6).

Organic acids and derivatives represented the predominant class (50%), including compounds such as betaine, creatine, pipecolic acid, and l‐threonine. This group mainly comprised amino acids, organic acids, and their derivatives, followed by organoheterocyclic compounds (17.1%), comprising nucleobases (uracil and cytosine), vitamins (pyridoxine and nicotinic acid) and indole‐related derivatives such as tryptophan derivatives. Organic nitrogen compounds (choline and spermidine) and organic oxygen compounds (muramic acid and l‐iditol, acetophenone) contributed equally to the metabolite set (7.1% each), whereas lipids and lipid‐like molecules (oleamide and palmitic amide) and nucleosides/nucleotides (uridine and adenosine) were present in moderate proportions (5.7% each). Minor yet functionally relevant groups were also detected, including alkaloids (trigonelline), and benzenoids (hordenine) (1.4% to 2.9%).

Pathway analysis further revealed significant overrepresentation of several core metabolic pathways (Fig. 5(B)). Among them, arginine and proline metabolism, glutathione metabolism, and purine metabolism showed the strongest enrichment, as indicated by larger and darker nodes in the network. These pathways are central to nitrogen metabolism and nucleotide biosynthesis, suggesting critical roles in stress response, growth, and energy metabolism. Additional pathways related to amino acid metabolism (e.g., valine, leucine, isoleucine, and lysine) were also enriched, indicating a comprehensive metabolic network consistent with the fungus's role in growth promotion and stress adaptation.

### Metabolic reprogramming of *C. aurantium* in response to *P. lilacinum*

#### Global metabolic discrimination revealed by OPLS‐DA

To study the impact of *P. lilacinum* treatment on the metabolome of *C. aurantium* seedlings, untargeted metabolomic profiling was performed on the leaves, stems, and roots of both inoculated and control groups using LC–MS/MS. A total of 158 metabolites were identified across major classes, including amino acids, phenolics, alkaloids, organic acids, carbohydrates, amines, and other compounds.

To interpret the metabolic changes induced by *P. lilacinum*, orthogonal partial least squares discriminant analysis (OPLS‐DA) was applied to the full dataset of identified metabolites from control and treated samples. The OPLS‐DA model was validated using regression parameters, yielding a high goodness of fit (*R* ^2^ *Y* = 0.996) and predictive ability (*Q* ^2^ *Y* = 0.99), indicating a robust and reliable model.

The OPLS‐DA score plots (Fig. 6) revealed clear, statistically significant clustering of control and treated samples for each plant part, highlighting distinct metabolic profiles between the groups. This separation accounted for 87.8% of the variation in leaves (Fig. 6(A)), 76.5% in stems (Fig. 6(B)), and 76.6% in roots (Fig. 6(C)). These high percentages of explained variance indicate that *P. lilacinum* induces a strong, systemic metabolic reprogramming throughout the host plant, moving beyond the site of inoculation to alter the chemical fingerprint of all vegetative organs significantly.

**Figure 6:**
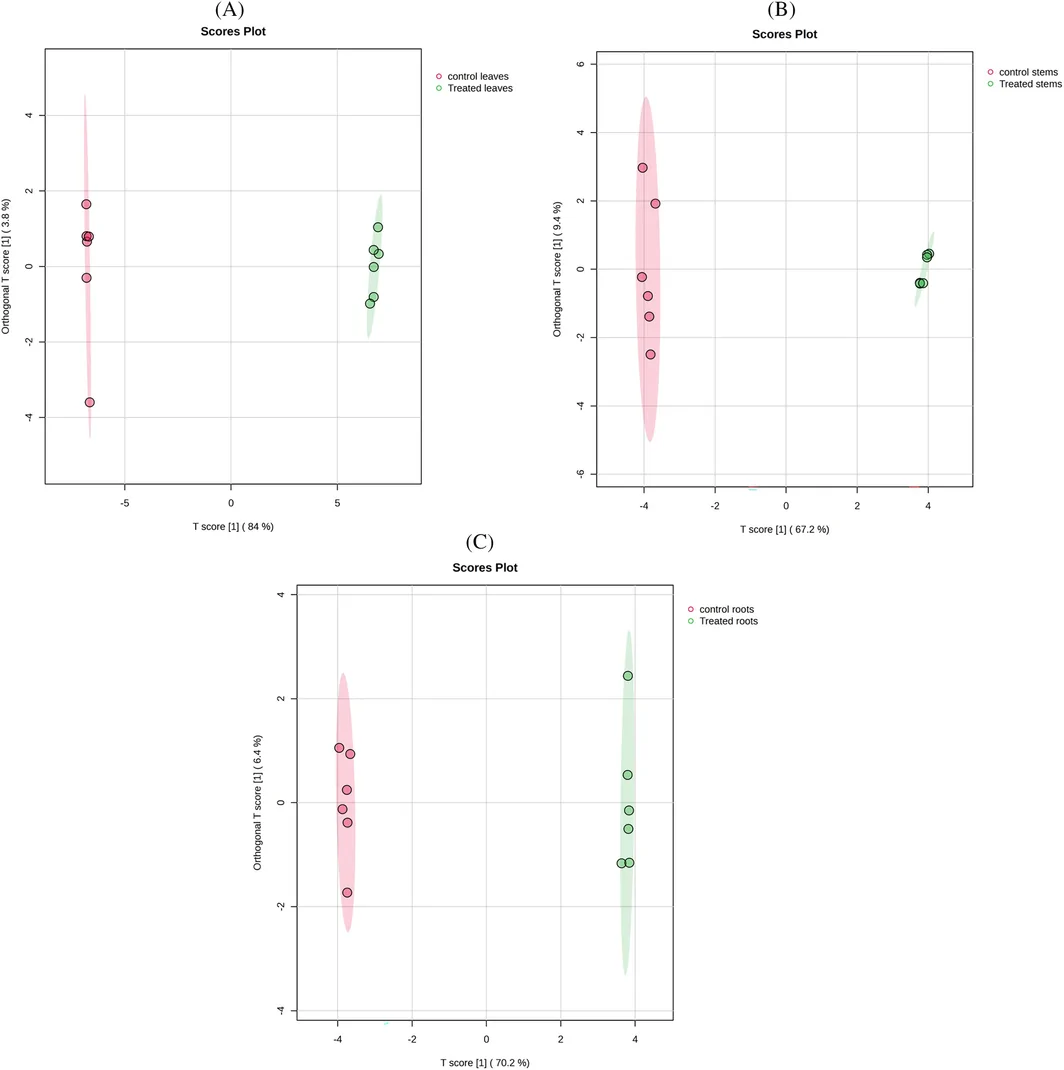
Orthogonal partial least squares discriminant analysis (OPLS‐DA) score plots showing (A) metabolic separation between control and *Purpureocillium lilacinum*‐treated *Citrus aurantium* samples in leaves, (B) stems, and (C) roots. Data were obtained by liquid chromatography–tandem mass spectrometry (LC–MS/MS) analysis. The OPLS‐DA scores plot is presented in electrospray ionization in positive mode (ESI+). All analyses were performed with six biological replicates (*n* = 6).

#### Discriminant metabolites identified by variable importance in projection analysis

Variable importance in projection scores were calculated for the OPLS‐DA models across all tissues to identify the specific metabolites driving the observed separations. The VIP analysis revealed distinct metabolite patterns among the leaves, stems, and roots of *C. aurantium* in response to *P. lilacinum* inoculation. In the leaves (Fig. 7(A)), quercetin was identified as the most significant biomarker (VIP = 2.3), showing a marked increase in treated samples, alongside other flavonoids and coumarins such as diosmetin, umbelliferone, and scoparone. The stems (Fig. 7(B)) were primarily characterized by the upregulation of phenolic compounds, specifically pyrogallol (VIP > 3.5) and isoferulic acid, whereas certain metabolites, such as ornithine, showed higher relative abundance in the control group. In the roots (Fig. 7(C)), l‐pyroglutamic acid emerged as the top discriminatory metabolite (VIP = 3.7) with higher levels in treated samples, followed by *trans*‐*Δ*2‐11‐methyl‐dodecenoic acid. Several defense‐related compounds and amino acid derivatives, such as kaempferol in leaves and hesperetin in roots, maintained high VIP scores (>1.0).

**Figure 7:**
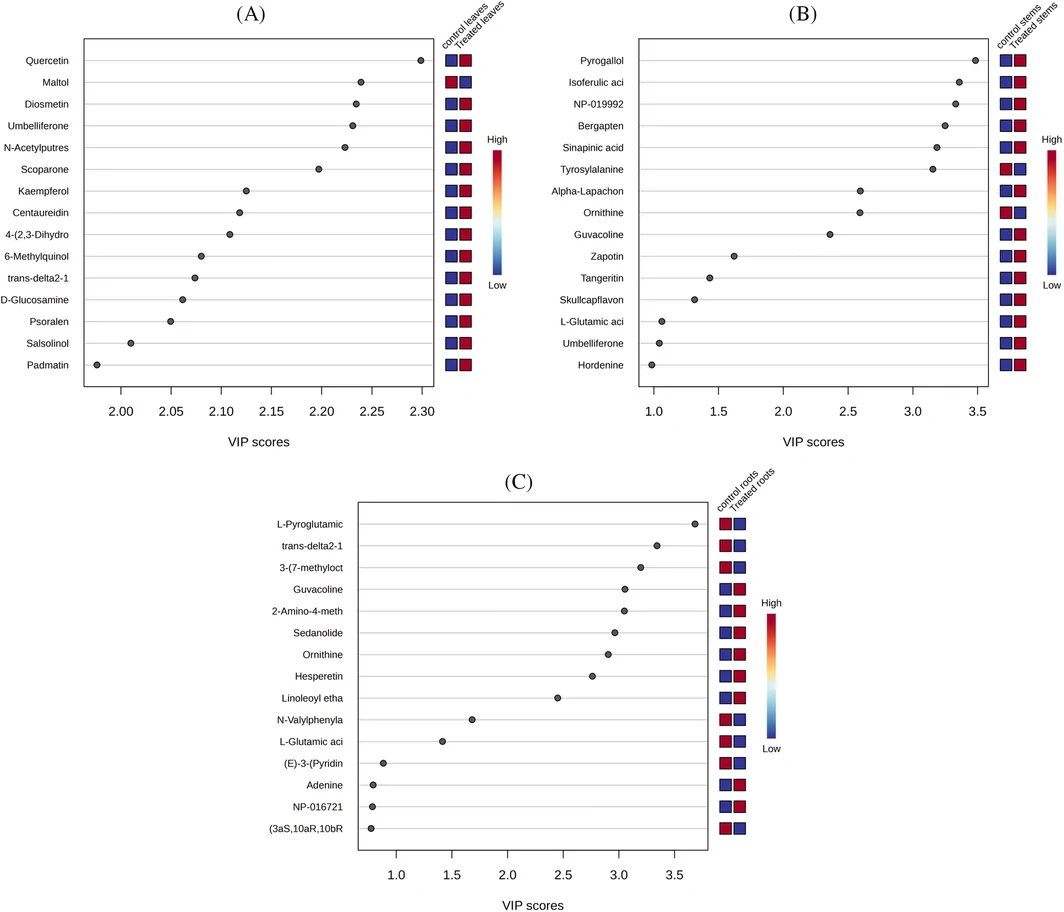
Variable importance in projection (VIP) scores of key metabolites differentiating *Purpureocillium lilacinum*‐treated and control *Citrus aurantium* plants in leaves (A), stems (B), and roots (C). Data were obtained by liquid chromatography–tandem mass spectrometry (LC–MS/MS) analysis in electrospray ionization in positive mode (ESI+). All analyses were performed with six biological replicates (*n* = 6).

#### Hierarchical clustering and systemic distribution of significantly regulated metabolites

The hierarchical clustering heatmaps revealed a profound and organ‐specific metabolic reprogramming in *C. aurantium* following *P. lilacinum* inoculation, with a clear separation between treated and control samples. Phenolic and polyphenolic compounds (Fig. 8(A)) were accumulated in leaves, including quercetin, kaempferol, diosmetin, scopoletin, scoparone, and salsolinol, indicating strong activation of the phenylpropanoid and flavonoid pathways associated with systemic acquired resistance and antioxidant protection. Amino acids (Fig. 8(B)) were predominantly enriched in roots and stems, notably proline, arginine, valine, and glutamine, reflecting enhanced nitrogen assimilation, osmotic adjustment, and source‐to‐sink nutrient redistribution supporting vegetative growth. Alkaloids, terpenoids, lactones and related metabolites (Fig. 8(C)) displayed a diverse induction pattern, with aromatic amines and indole‐related compounds accumulating in leaves and roots, consistent with stimulation of induced systemic resistance and antimicrobial and insect‐deterrent activity. Finally, lipid profiles (Fig. 8(D)) showed coordinated modulation of fatty acids and signaling lipids such as *α*‐eleostearic acid and *N*‐acylethanolamines, highlighting membrane remodeling and lipid‐mediated defense signaling.

**Figure 8:**
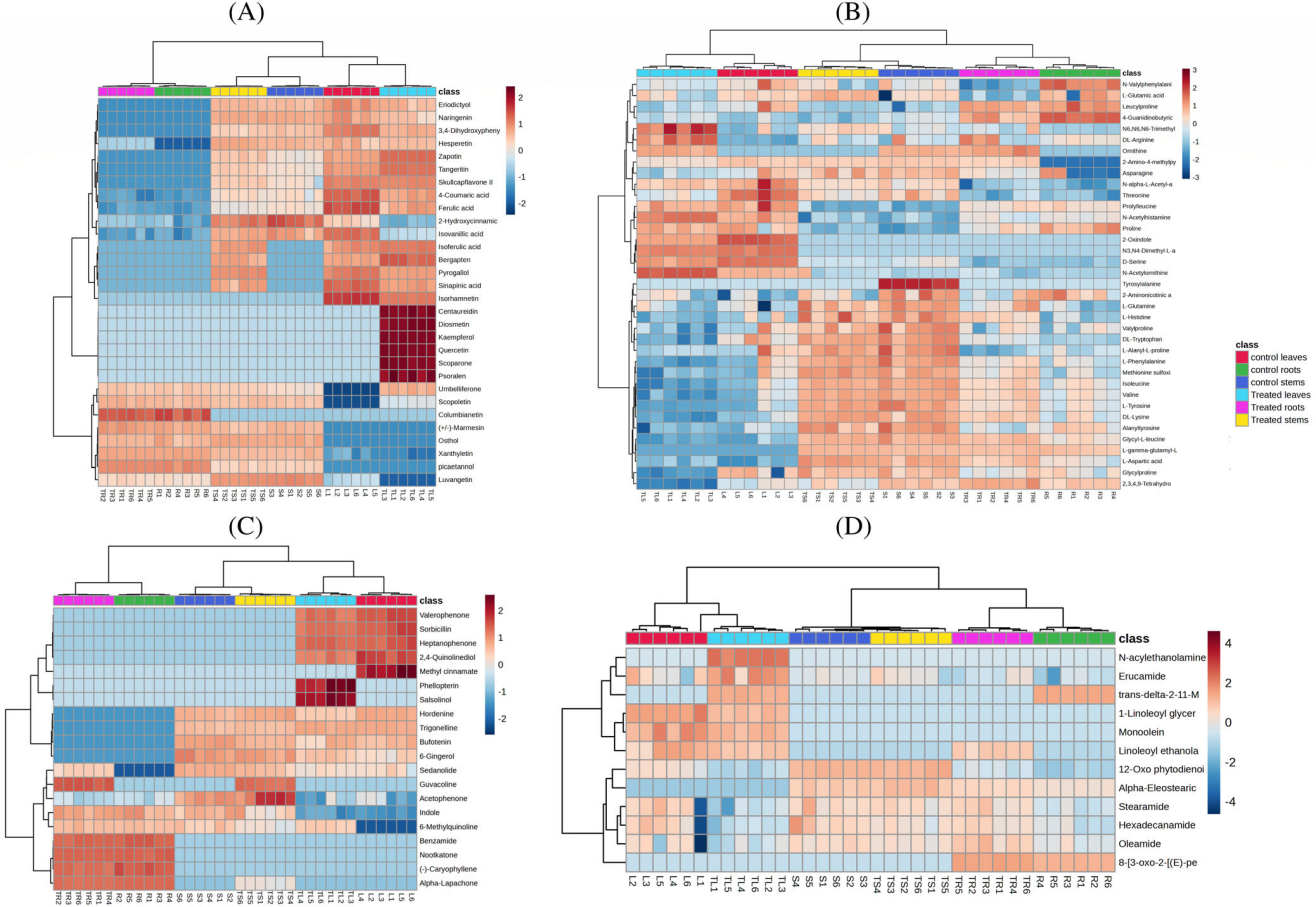
Hierarchical clustering heatmaps showing organ‐specific modulation of metabolite classes in *Citrus aurantium* leaves, stems, and roots following *Purpureocillium lilacinum* inoculation: (A) phenolics and polyphenols, (B) amino acids, (C) alkaloids/terpenoids/lactones, and (D) lipids. Data were obtained by liquid chromatography–tandem mass spectrometry (LC–MS/MS) analysis in electrospray ionization positive mode (ESI+). The color scale represents normalized intensity values, ranging from low (blue) to high (red). Only significantly different metabolites are shown (*P* < 0.05, variable importance in projection (VIP) > 1.0). All analyses were performed with six biological replicates (*n* = 6).

#### Volcano plot analysis of metabolite modulation across plant tissues

Volcano plot analysis (Fig. 9), based on adjusted *P*‐values (*P* < 0.05) and fold‐change thresholds (|log_2_FC| ≥ 1), revealed significant modulation in metabolite abundance. In leaves (Fig. 9(A)), 61 metabolites were significantly reprogrammed upon treatment, including 31 upregulated and 30 downregulated compounds. Upregulated metabolites mainly comprised phenolic compounds, coumarins, alkaloids, amino acid derivatives, and flavonoids, with the highest increases observed for quercetin (log_2_FC = 9.07), diosmetin (8.42), scoparone (8.03), umbelliferone (8.38), and kaempferol (7.26). These high values reflect clear biological responses, driven primarily by the near‐absence of these compounds in control samples and their strong accumulation following treatment.

**Figure 9:**
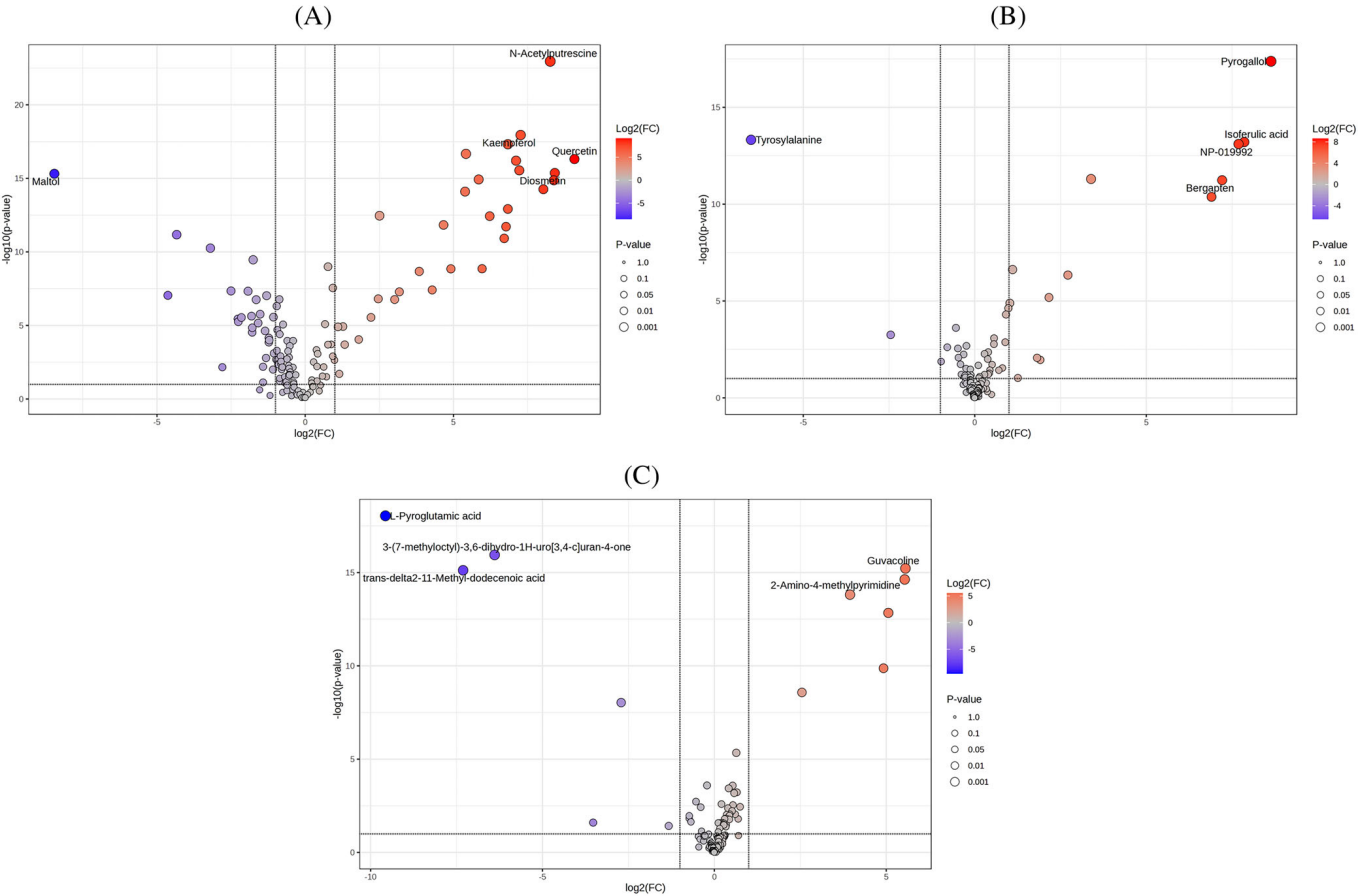
Volcano plots of differentially accumulated metabolites between control and *Purpureocillium lilacinum*‐treated plants in (A) leaves, (B) stems, and (C) roots. The *x*‐axis represents log_2_ fold change and the *y*‐axis represents ‐log₁₀ adjusted *P*‐values. Data were obtained by liquid chromatography–tandem mass spectrometry (LC–MS/MS) analysis in electrospray ionization positive mode (ESI+). Thresholds for significance were set at *P* < 0.05 and |log_2_FC| ≥ 1. All analyses were performed with six biological replicates (*n* = 6).

Conversely, downregulated metabolites comprised amino acid derivatives, and small organic acids, with the most pronounced decreases for maltol (log_2_FC = −8.46), 2‐oxindole (−4.63), methyl cinnamate (−4.33), luvangetin (−3.20), and 2,3,4,9‐tetrahydro‐1*H*‐*β*‐carboline‐3‐carboxylic acid (−2.50).

Fifteen metabolites were affected significantly in stems (Fig. 9(B)), including 13 upregulated and two downregulated compounds. The strongest increases were observed for pyrogallol (8.64), isoferulic acid (7.85), NP‐019992 (7.69), bergapten (7.21), and sinapinic acid (6.90), whereas tyrosylalanine (−6.52) and ornithine (−2.45) showed the largest decreases.

In roots (Fig. 9(C)), 12 metabolites were significantly modulated, including upregulation of guvacoline (5.56), 2‐amino‐4‐methylpyrimidine (5.54), sedanolide (5.06), ornithine (4.92), hesperetin (3.95), and linoleoyl ethanolamide (2.55), and downregulation of l‐pyroglutamic acid (−9.57), *trans‐*delta‐2‐11‐methyl‐dodecenoic acid (−7.31), 3‐(7‐methyloctyl)‐3,6‐dihydro‐1*H*‐furo[3,4‐*c*]uran‐4‐one (−6.39), l‐glutamic acid (−3.53), *N*‐valylphenylalanine (−2.71), and (*E*)‐3‐(pyridin‐4‐ylmethylene)chroman‐4‐one (−1.33).

## DISCUSSION

The integrated metabolomic profiling of *P. lilacinum* and its citrus host, *C. aurantium*, reveals that this fungal symbiont operates through a concerted tripartite strategy to enhance plant performance. This strategy encompasses (1) the direct delivery of growth‐promoting and antagonistic metabolites from the fungal arsenal, (2) the initiation of a metabolic dialogue that systemically reprograms host primary metabolism, and (3) the elicitation of a spatially organized plant secondary defense metabolism.

The first layer of this strategy involves direct metabolic contributions from the fungal arsenal. *Purpureocillium lilacinum* first interacts with its host through intrinsic functional traits that directly enhance nutrient acquisition and plant development. In the context of alkaline, phosphorus‐fixing soils, typical of citrus orchards,^42^ the fungus demonstrated a strong capacity to solubilize inorganic phosphate. Its prolific and time‐dependent production of siderophores improves iron nutrition while competitively excluding pathogens.^29^, ^30^ The fungus showed a pronounced ability to synthesize IAA from l‐tryptophan, with peak accumulation at 168 h. Metabolomic analysis confirmed the presence of IAA and related indole derivatives, including indoleacrylic acid and d‐tryptophan, indicating an active indole metabolic pathway. Given the central regulatory role of auxins in plant development,^43^, ^44^ fungal‐derived IAA production provides a mechanistic explanation for the enhanced plant height, shoot biomass, and leaf number observed in inoculated citrus plants.

This targeted auxin production is a key component of a far more extensive metabolic repertoire. Indeed, the effectiveness of *P. lilacinum* is rooted in its diverse metabolic arsenal; its internal metabolomic profile underscores its biochemical versatility, revealing 70 identified metabolites distributed across nine distinct chemical classes. This chemically diverse profile reflects a strong metabolic plasticity and ecological competence in the rhizosphere, highlighting the capacity of the fungus to function as a multifunctional biological agent. The detection of osmoprotective and signaling metabolites such as betaine, proline, and pipecolic acid highlights a dual role in stress tolerance and immune priming. Proline and betaine contribute to osmotic balance, membrane stability, and redox homeostasis,^45^, ^46^ while pipecolic acid is a key regulator of systemic acquired resistance (SAR),^47^, ^48^ supporting the induction of plant‐wide defense preparedness.

The stimulation of primary metabolism was further reflected by a marked increase in photosynthetic pigments with total chlorophyll content increasing more than threefold in citrus. This response aligns with the fungal production of essential vitamins, including pyridoxine (vitamin B_6_), the precursor of pyridoxal‐5′‐phosphate (PLP), a universal cofactor for amino acid metabolism and nitrogen assimilation.^49^ Beyond its general metabolic role, PLP is directly implicated in chlorophyll biosynthesis, as PLP‐dependent enzymes catalyze key steps in the formation of *δ*‐aminolevulinic acid (ALA), the universal precursor of all tetrapyrrole compounds, including chlorophyll.^50^ Similarly, nicotinic acid (vitamin B_3_) serves as the precursor of NAD^+^ and NADP^+^, the central redox carriers that drive primary carbon metabolism and photosynthesis – including the NADPH‐dependent reduction of protochlorophyllide, a key step in chlorophyll biosynthesis.^51^, ^52^, ^53^

Beyond growth promotion, the fungal metabolome revealed a direct biocontrol capacity, characterized by metabolites with insect‐modulating and antimicrobial properties, underscoring a multipronged biocontrol strategy. For instance, detected acetophenone and related aromatic compounds can influence insect behavior as repellents or oviposition deterrents. Experimental studies have demonstrated reduced attraction and oviposition of *Drosophila suzukii* in the presence of acetophenone.^54^ Structurally related derivatives have also been shown to inhibit acetylcholinesterase activity, disrupting neural transmission and feeding in pests like *Tribolium castaneum*.^55^, ^56^

The detection of specific alkaloids further reinforces a direct insecticidal dimension. Arecoline was detected in the fungal metabolomic profile. This compound has been reported primarily in plant systems, particularly in *Areca catechu*.^57^ As far as the authors of the current study are aware, and based on their literature search across major databases (including PubMed, Scopus, and Google Scholar), arecoline has not been documented previously in pure fungal metabolomic profiles. It exhibits insecticidal activity against *Plutella xylostella* larvae through enzymatic inhibition that disrupts physiological processes.^57^ Its presence suggests *P. lilacinum* may suppress pests by direct biochemical interference.

Similarly, hordenine, detected in the fungal secretome, acts as an effective antifeedant against lepidopteran and orthopteran pests.^58^ This alkaloid also demonstrates a dual function by upregulating pathogenesis‐related (PR) protein genes in plants, linking direct herbivore deterrence with the activation of plant immune responses.

Polyamines, particularly spermidine, represent an additional layer of protection. These molecules are central regulators of stress signaling and antioxidant capacity, enhancing tolerance to both abiotic and biotic challenges.^59^, ^60^ Collectively, the presence of aromatics, alkaloids, and polyamines demonstrates that *P. lilacinum* deploys a multi‐target biocontrol strategy combining direct pest suppression with host defense reinforcement.

The impact of this fungal arsenal extends beyond direct provision, triggering a systemic metabolic reprogramming in the host plant. Multivariate analyses revealed a pronounced metabolic separation between treated and control plants across all tissues (leaves, stems, roots), indicating a whole‐plant physiological shift – a hallmark of beneficial plant‐microbe interactions where the symbiont modulates host metabolism for mutual benefit.^61^, ^62^ This reprogramming is driven by active metabolic cross‐talk. Key metabolites, including proline, arginine, branched‐chain amino acids, phenylalanine, pipecolic acid, and lipid‐derived signals, were coordinately regulated in both the fungus and the inoculated plant. This overlap suggests fungal metabolites or induced host pathways act as mobile signals that reshape plant metabolic networks, a mechanism well‐documented in other symbiotic associations.^63^, ^64^

The systemic nature of this plant‐fungal interaction is exemplified by the spatial reprogramming of key amino acids. Following inoculation, proline accumulated markedly in leaves and stems while decreasing in roots. This source‐to‐sink translocation mobilizes root‐synthesized proline to aerial tissues, where its roles in osmotic adjustment, membrane stabilization, and redox homeostasis^65^, ^66^ directly support the observed enhancements in photosynthesis and growth under stress. This response is coordinated with the accumulation of other osmoprotectants like betaine, collectively priming the plant for resilience.

The accumulation of ornithine and its precursor, arginine, concurrently activates a central nitrogen hub for defense signaling. Arginine, which significantly increased across all plant tissues, serves as a major nitrogen reserve and a precursor for polyamines and nitric oxide – key regulators of stress signaling and development.^67^ The tissue‐specific accumulation of ornithine in the leaves and roots indicates its rapid channeling from this arginine pool into polyamine biosynthesis, a pathway critical for cell division and defense.^68^, ^69^

The discovery that ornithine, arginine, and specific polyamine derivatives (*N* ^8^‐acetylspermidine and *N*‐acetylputrescine) are present in both the fungal metabolome and the inoculated plant provides definitive evidence of active metabolic cross‐talk.^70^ This shared metabolic landscape reveals an intertwined physiology where fungal signals or metabolites stimulate and integrate with the plant's own nitrogen and defense metabolism. The tight interconnection between the arginine, ornithine, and proline pathways^71^, ^72^ underscores a coordinated strategy: nitrogen is diverted into the synthesis of polyamines for defense signaling and proline for stress protection. Through this integrated network, polyamines stabilize membranes and modulate defense genes,^73^ while proline safeguards cellular function, jointly enhancing the plant's adaptive capacity.

Building upon the reprogramming of key amino acid pathways, the interaction is further defined by a shared metabolic focus on tryptophan. The detection of specific indole derivatives – indoleacrylic acid, 5‐hydroxyindoleacetic acid, and dl‐tryptophan – in both the fungal metabolome and inoculated plant tissues underscores the centrality of tryptophan metabolism in this cross‐talk. The presence of shared intermediates indicates that *P. lilacinum* either contributes to this metabolic pool or actively stimulates the plant's own biosynthetic networks.

Critically, this shared pathway has direct phenotypic consequences, as tryptophan metabolism represents a fundamental biochemical channel through which the fungus influences both the growth and defense physiology of its host.

Extending beyond primary metabolism, *P. lilacinum* inoculation triggered a profound reprogramming of citrus secondary metabolism, highlighting a critical layer of fungus‐mediated biocontrol. This was marked by a systemic accumulation of phenolic compounds, with a pronounced activation of the phenylpropanoid and flavonoid pathways. Key metabolites such as quercetin, kaempferol, scoparone, and umbelliferone were enriched significantly. Maltol, a naturally occurring flavor compound with documented antioxidant and antimicrobial properties, was also detected among the downregulated metabolites in inoculated leaves. Although not among the most frequently reported metabolites in *Citrus* species, its presence in citrus peel tissue has been previously documented by GC–MS analysis of *Citrus reticulata* ethanolic extracts,^74^ supporting the validity of its identification in the present study. Its downregulation may reflect a metabolic reallocation of shared precursors toward the enhanced biosynthesis of flavonoids and coumarins observed in inoculated tissues.

Increased flavonoid levels can sometimes reflect a general response to biotic stress but the concurrent and significant enhancement in biomass, plant height, and photosynthetic pigments, sustained 1 year after inoculation, strongly supports a specific and durable priming of host defenses rather than a transient stress reaction. Nevertheless, while this 1 year time point suggests stable priming, monitoring intermediate time points would be required to provide definitive proof of the long‐term metabolic kinetics and completely rule out non‐specific stress fluctuations. This secondary metabolic shift orchestrates a dual enhancement of defense and growth. Flavonoids like quercetin and kaempferol strengthen antioxidant defenses and fine‐tune photosynthesis under stress,^75^ while also modulating auxin transport.^76^ Coumarins (e.g., scoparone, scopoletin) further bolster antimicrobial defenses. Beyond their direct protective role, these compounds contribute to iron chelation and redox balance, directly supporting chloroplast function.^77^ Their leaf‐specific accumulation provides a mechanistic basis for the significant increases in chlorophyll and carotenoids, directly linking secondary metabolism to enhanced photosynthetic performance.

This reprogramming exhibits precise spatial organization for targeted defense. In stems, the accumulation of sinapinic acid, isoferulic acid, pyrogallol, and bergapten indicates reinforced structural and chemical barriers. Sinapinic and isoferulic acids are precursors for lignin biosynthesis, strengthening cell walls against pathogen spread.^78^ Pyrogallol may also establish antimicrobial chemical barriers in vascular tissues, and the phototoxic furanocoumarin bergapten provides direct biocontrol activity against insects and pathogens.^79^, ^80^

## CONCLUSION

The integrated metabolomic profile of *P. lilacinum* and its citrus host reveals a sophisticated, multilayered mode of action that synergizes direct stimulation with systemic priming. This interaction operates through three complementary mechanisms: first, the fungus directly enhances growth vigor by solubilizing nutrients, modulating hormones like IAA, and providing essential metabolites, improving root architecture and biomass; second, it systemically primes plant defenses by inducing and sharing key metabolites such as pipecolic acid and polyamines, enabling a faster, stronger immune response to biotic challenges; and third, it employs a direct biocontrol arsenal, as its metabolome contains compounds like arecoline and acetophenone with documented antimicrobial and insect‐modulating properties for direct antagonism. This tripartite functionality, simultaneously boosting host health and activating multifaceted defenses, provides a particularly valuable and resilient strategy for sustainable citrus cultivation under rising pest pressure and reduced pesticide reliance.

Consistent orchard‐level performance requires addressing key challenges, such as environmental variability, competition with native soil microbiota, and host genotype specificity, to translate these findings effectively. The first and indispensable requirement is robust colonization capacity and host compatibility, as successful endophytic establishment is a prerequisite for all downstream functional effects. The second level includes core plant growth‐promoting traits, namely IAA, siderophore, and phosphate‐solubilizing activity, which together determine the ability of a strain to enhance nutrient acquisition and stimulate early plant development under field conditions. The third level concerns functional specialization, where strains can be prioritized further based on their secondary metabolite repertoire – including osmoprotectants, immune‐priming compounds, and antimicrobial or insecticidal metabolites – depending on the dominant abiotic or biotic constraints in the target environment.

In addition to strain prioritization, the metabolomics‐guided framework developed in this study provides practical tools for evaluating inoculation success using metabolite‐based biomarkers rather than relying solely on phenotypic traits. Successful colonization and functional activation can be inferred from a coordinated metabolic signature characterized by the redistribution of proline from roots to aerial tissues, enhanced arginine accumulation, and the increased abundance of key defense and growth‐associated flavonoids and coumarins, particularly quercetin, kaempferol, and scoparone. To monitor these dynamics effectively in citrus production, a strategic sampling timeline adapted to the perennial growth kinetics of woody hosts is recommended. An initial evaluation should be performed at 2 to 3 months post‐inoculation (ideally coinciding with the first seasonal vegetative flush) to verify successful endophytic establishment through early biomarkers, such as coumarin and flavonoid accumulation. A second monitoring window is recommended at 6 months post‐inoculation or during subsequent peak pest/pathogen pressure periods to assess long‐term systemic priming, as evidenced by sustained proline redistribution and enhanced arginine profiles.

Metabolomic responses were evaluated at a single post‐inoculation time point in the present study; however, these biomarkers provide a robust foundation for future research aimed at optimizing field monitoring strategies. Temporal validation under field conditions will be required to determine the most informative sampling intervals for assessing colonization dynamics, persistence, and long‐term functional stability of the symbiosis. Such studies will be essential to translate metabolomics from a descriptive analytical tool into a predictive framework for monitoring bioinoculant performance in agricultural systems.

The metabolomics‐guided approach presented here extends beyond mechanistic insight, offering a practical and integrative platform for the selection, evaluation, and future deployment of *P. lilacinum*‐based bioinoculants. This framework contributes to a more rational and evidence‐based development of microbial solutions for sustainable citrus production systems.

## Supporting information


**Figure S1.** Agarose gel electrophoresis of PCR products confirming Citrus aurantium colonization by Purpureocillium lilacinum. Lane M: 1 kb DNA ladder; Lanes 1–6: colonized plants showing amplification of the expected 123 bp fragment; Lane 7: negative control (no template DNA).

**Table S1.** Metabolites identified in Purpureocillium lilacinum by LC–MS/MS (positive ionization mode, ES^+^; *n* = 6) and their significant abundance change in Citrus aurantium leaves, stems, and roots following fungal inoculation (Treated *vs*. Control; Student's *t*‐test, *P* < 0.05, FDR‐corrected). All compounds correspond to MSI Level 2 (putative annotation based on spectral library MS/MS match).

## Data Availability

The data that supports the findings of this study are available in the supplementary material of this article.
