# Supplementary material for: Beyond a dual role: how Purpureocillium lilacinum reprograms citrus metabolism for integrated biocontrol and growth promotion
Source: J Sci Food Agric. 2026 Aug 13;106(15):9778–93. doi: 10.1002/jsfa.70954 (PMC13743398; doi:10.1002/jsfa.70954)
Supplement: Supplementary file 1 [file JSFA-106-9778-s001.docx]

**Supplementary Figure 1.** Agarose gel electrophoresis of PCR products confirming Citrus aurantium colonization by Purpureocillium lilacinum. Lane M: 1 kb DNA ladder; Lanes 1-6: colonized plants showing amplification of the expected 123 bp fragment; Lane 7: negative control (no template DNA).
