# Supplementary material for: Beyond a dual role: how Purpureocillium lilacinum reprograms citrus metabolism for integrated biocontrol and growth promotion
Source: J Sci Food Agric. 2026 Aug 13;106(15):9778–93. doi: 10.1002/jsfa.70954 (PMC13743398; doi:10.1002/jsfa.70954)
Supplement: Supplementary file 2 [file JSFA-106-9778-s002.docx]

Table S1. Metabolites identified in Purpureocillium lilacinum by LC-MS/MS (positive ionization mode, ES⁺; n = 6) and their significant abundance change in Citrus aurantium leaves, stems, and roots following fungal inoculation (Treated vs. Control; Student's t-test, p < 0.05, FDR-corrected). All compounds correspond to MSI Level 2 (putative annotation based on spectral library MS/MS match).

| RT | Compounds | Formula | Adduits | Ion m/z | Identification level | Leaves | Stems | Roots |
| --- | --- | --- | --- | --- | --- | --- | --- | --- |
| 0.137 | Spermidine | C7H19N3 | [M+H]+1 | 145.1577943 | Level 2 | ND | ND | ND |
| 1.198 | Glucose 1-phosphate | C6H13O9P | [M+H]+1 | 260.02942 | Level 2 | Significant change | NS | NS |
| 1.487 | L-Iditol | C6H14O6 | [M+H]+1 | 182.078935 | Level 2 | ND | ND | ND |
| 1.615 | D-Serine | C3H7NO3 | [M+H]+1 | 105.0428943 | Level 2 | Significant change | NS | NS |
| 1.634 | L-Cystathionine | C7H14N2O4S | [M+H]+1 | 222.0671586 | Level 2 | ND | ND | ND |
| 1.686 | Muramic acid | C9H17NO7 | [M+H]+1 | 251.1001933 | Level 2 | Significant change | NS | NS |
| 1.698 | N-Acetylneuraminic acid | C11H19NO9 | [M+H]+1 | 309.105495 | Level 2 | ND | ND | ND |
| 1.708 | Choline | C5H13NO | [M+H]+1 | 103.1000043 | Level 2 | ND | ND | ND |
| 1.721 | L-Threonine | C4H9NO3 | [M+H]+1 | 119.0583857 | Level 2 | Significant change | NS | NS |
| 1.741 | Glutamic acid | C5H9NO4 | [M+H]+1 | 147.053097 | Level 2 | NS | NS | NS |
| 1.798 | Saccharopine | C11H20N2O6 | [M+H]+1 | 276.131747 | Level 2 | ND | ND | ND |
| 1.845 | Betaine | C5H11NO2 | [M+H]+1 | 117.079133 | Level 2 | NS | ND | ND |
| 1.953 | Proline | C5H9NO2 | [M+H]+1 | 115.0635611 | Level 2 | NS | NS | NS |
| 2.055 | Ornithine | C5H12N2O2 | [M+H]+1 | 132.08992 | Level 2 | Significant change | Significant change | Significant change |
| 2.142 | Histidine | C6H9N3O2 | [M+H]+1 | 155.0694043 | Level 2 | Significant change | NS | Significant change |
| 2.143 | Pipecolic acid | C6H11NO2 | [M+H]+1 | 129.0790025 | Level 2 | Significant change | NS | NS |
| 2.162 | Trigonelline | C7H7NO2 | [M+H]+1 | 137.047642 | Level 2 | NS | Significant change | ND |
| 2.244 | N2-Acetylornithine | C7H14N2O3 | [M+H]+1 | 174.1003543 | Level 2 | ND | ND | ND |
| 2.247 | Dimethylglycine | C4H9NO2 | [M+H]+1 | 103.063 | Level 2 | ND | ND | ND |
| 2.377 | DL-Arginine | C6H14N4O2 | [M+H]+1 | 174.1115629 | Level 2 | Significant change | Significant change | Significant change |
| 2.385 | Maltol | C6H6O3 | [M+H]+1 | 126.0317833 | Level 2 | Significant change | ND | ND |
| 2.460 | Creatine | C4H9N3O2 | [M+H]+1 | 131.0694786 | Level 2 | ND | ND | ND |
| 2.718 | Pyroglutamic acid | C5H7NO3 | [M+H]+1 | 129.0426186 | Level 2 | Significant change | NS | sig |
| 2.718 | Uracil | C4H4N2O2 | [M+H]+1 | 112.0275129 | Level 2 | Significant change | NS | NS |
| 2.731 | Imidazolelactic acid | C6H8N2O3 | [M+H]+1 | 156.053442 | Level 2 | ND | ND | ND |
| 2.765 | N6-Trimethyl-L-lysine | C9H20N2O2 | [M+H]+1 | 188.1523229 | Level 2 | Significant change | Significant change | Significant change |
| 2.794 | Nicotinic acid | C6H5NO2 | [M+H]+1 | 123.0321357 | Level 2 | ND | ND | ND |
| 2.798 | Aminoadipic acid | C6H11NO4 | [M+H]+1 | 161.0686988 | Level 2 | ND | ND | ND |
| 2.798 | 5-Aminopentanoic acid | C6H11NO4 | [M+H]+1 | 161.0686988 | Level 2 | ND | ND | ND |
| 3.019 | L-Valine | C5H11NO2 | [M+H]+1 | 117.0791729 | Level 2 | Significant change | NS | Significant change |
| 3.147 | L-Carnitine | C7H15NO3 | [M+H]+1 | 161.1051257 | Level 2 | ND | ND | ND |
| 3.211 | N6-Acetyl-L-lysine | C8H16N2O3 | [M+H]+1 | 188.116002 | Level 2 | ND | ND | ND |
| 3.552 | Cytidine | C9H13N3O5 | [M+H]+1 | 243.0852729 | Level 2 | NS | NS | NS |
| 3.568 | Hypoxanthine | C5H4N4O | [M+H]+1 | 136.038475 | Level 2 | ND | ND | ND |
| 3.581 | Inosine | C10H12N4O5 | [M+H]+1 | 268.0804675 | Level 2 | ND | ND | ND |
| 3.806 | Guanine | C5H5N5O | [M+H]+1 | 151.0493414 | Level 2 | Significant change | NS | NS |
| 3.920 | N-alpha-Acetyl-L-arginine | C8H16N4O3 | [M+H]+1 | 216.12211 | Level 2 | ND | ND | ND |
| 3.982 | Glycylproline | C7H12N2O3 | [M+H]+1 | 172.084775 | Level 2 | Significant change | NS | NS |
| 4.099 | Arecoline | C8H13NO2 | [M+H]+1 | 155.0946125 | Level 2 | ND | ND | ND |
| 4.179 | Adenine | C5H5N5 | [M+H]+1 | 135.0542989 | Level 2 | Significant change | Significant change | Significant change |
| 4.183 | Adenosine | C10H13N5O4 | [M+H]+1 | 267.0966114 | Level 2 | NS | NS | NS |
| 4.299 | 5-Hydroxylysine | C9H8O3 | [M+H]+1 | 164.0473229 | Level 2 | ND | ND | ND |
| 4.299 | 2-Hydroxycinnamic acid | C9H8O3 | [M+H]+1 | 164.04733 | Level 2 | Significant change | NS | Significant change |
| 4.300 | 2-Hydroxyphenylalanine | C9H11NO3 | [M+H]+1 | 181.0738733 | Level 2 | ND | ND | ND |
| 4.493 | Alanylproline | C8H14N2O3 | [M+H]+1 | 186.10039 | Level 2 | ND | ND | ND |
| 4.639 | Isoleucine | C6H13NO2 | [M+H]+1 | 131.0946557 | Level 2 | Significant change | NS | Significant change |
| 4.719 | 2-O-Methyladenosine | C11H15N5O4 | [M+H]+1 | 281.112179 | Level 2 | ND | ND | ND |
| 4.807 | Pyridoxine | C8H11NO3 | [M+H]+1 | 169.0738571 | Level 2 | Significant change | NS | NS |
| 4.871 | L-Norleucine | C6H13NO2 | [M+H]+1 | 131.0946514 | Level 2 | Significant change | NS | NS |
| 5.388 | N-Acetyl-Leu | C8H15NO3 | [M+H]+1 | 173.105072 | Level 2 | ND | ND | ND |
| 5.402 | Phenylalanine | C9H11NO2 | [M+H]+1 | 165.0789243 | Level 2 | Significant change | NS | Significant change |
| 5.510 | L-Acetylcarnitine | C9H17NO4 | [M+H]+1 | 203.115705 | Level 2 | ND | ND | ND |
| 5.649 | 5-Hydroxyindoleacetic acid | C10H9NO3 | [M+H]+1 | 191.05822 | Level 2 | ND | ND | ND |
| 6.026 | Acetophenone | C8H8O | [M+H]+1 | 120.0577367 | Level 2 | Significant change | Significant change | NS |
| 6.123 | Valylproline | C10H18N2O3 | [M+H]+1 | 214.13168 | Level 2 | Significant change | Significant change | NS |
| 7.088 | Indoleacrylic acid | C11H9NO2 | [M+H]+1 | 187.06331 | Level 2 | Significant change | NS | Significant change |
| 7.093 | D-Tryptophan | C11H12N2O2 | [M+H]+1 | 204.0898514 | Level 2 | Significant change | NS | Significant change |
| 7.528 | Glycylleucine | C8H16N2O3 | [M+H]+1 | 188.1160757 | Level 2 | ND | ND | ND |
| 7.634 | 6-Methylquinoline | C10H9N | [M+H]+1 | 143.0734886 | Level 2 | Treated Only | Significant change | Significant change |
| 7.755 | Hexanoylcarnitine | C13H25NO4 | [M+H]+1 | 259.17821 | Level 2 | ND | ND | ND |
| 7.779 | Hordenine | C10H15NO | [M+H]+1 | 165.1154175 | Level 2 | Significant change | Significant change | ND |
| 8.888 | N8-Acetylspermidine | C9H21N3O | [M+H]+1 | 187.1684633 | Level 2 | Treated Only | ND | ND |
| 9.014 | Leucylproline | C11H20N2O3 | [M+H]+1 | 228.14724 | Level 2 | Significant change | NS | NS |
| 11.154 | Valylphenylalanine | C14H20N2O3 | [M+H]+1 | 264.147294 | Level 2 | Significant change | NS | Significant change |
| 16.299 | 9-Oxo-10(E),12(E)-octadecadienoic acid | C18H30O3 | [M+H]+1 | 294.2190333 | Level 2 | ND | ND | ND |
| 16.428 | Linoleoyl ethanolamide | C20H37NO2 | [M+H]+1 | 323.2817971 | Level 2 | Significant change | ND | Treated Only |
| 16.583 | Palmitic amide | C18H37NO2 | [M+H]+1 | 299.282018 | Level 2 | ND | ND | ND |
| 16.585 | Palmitoylethanolamide | C18H37NO2 | [M+H]+1 | 299.28198 | Level 2 | ND | ND | ND |
| 16.936 | Stearoylethanolamide | C20H41NO2 | [M+H]+1 | 327.3126775 | Level 2 | ND | ND | ND |
| 16.955 | Oleamide | C18H35NO | [M+H]+1 | 281.27133 | Level 2 | Significant change | NS | NS |

Legend: Significant change: p < 0.05, FDR-corrected, Treated vs. Control (Student's t-test); NS: no significant change; ND: not detected.
